# A new family of distributions using a trigonometric function: Properties and applications in the healthcare sector

**DOI:** 10.1016/j.heliyon.2024.e29861

**Published:** 2024-04-22

**Authors:** Omalsad Hamood Odhah, Huda M. Alshanbari, Zubair Ahmad, Faridoon Khan, Abd al-Aziz Hosni El-Bagoury

**Affiliations:** a Department of Mathematical Sciences, College of Science, Princess Nourah bint Abdulrahman University, P.O. Box 84428, Riyadh 11671, Saudi Arabia; b Department of Statistics, Quaid-i-Azam University, Islamabad 44000, Pakistan; c Pakistan Institute of Development Economics, Islamabad 44000, Pakistan; d Basic Sciences Department, Higher Institute of Engineering and Technology, EL-Mahala EL-Kobra, Egypt

**Keywords:** Trigonometric function, Sine function, Weibull distribution, Monte Carlo simulation, Medical data sets, Statistical modeling

## Abstract

Probability distributions play a pivotal and significant role in modeling real-life data in every field. For this activity, a series of probability distributions have been introduced and exercised in applied sectors. This paper also contributes a new method for modeling continuous data sets. The proposed family is called the exponent power sine-*G* family of distributions. Based on the exponent power sine-*G* method, a new model, namely, the exponent power sine-Weibull model is studied. Several mathematical properties such as quantile function, identifiability property, and $r^{th}$ moment are derived. For the exponent power sine-*G* method, the maximum likelihood estimators are obtained. Simulation studies are also presented. Finally, the optimality of the exponent power sine-Weibull model is shown by taking two applications from the healthcare sector. Based on seven evaluating criteria, it is demonstrated that the proposed model is the best competing distribution for analyzing healthcare phenomena.

## 1 Introduction

It is a clear crystal fact that no specific probabilistic model can perform better in all situations to provide the optimal fit to real phenomena (i.e., real-life data sets). Therefore, a significant number of statistical sectors always seek to develop new probability distributions with novel criteria. In most cases, the new probability distributions (or updated versions of existing distributions) may adequately fit the real phenomena than existing or contender models. This fact has inspired practitioner to explore new probability distributions with applications in various fields of life. For brief reviews of the development of new probability distributions [1], [2], [3], [4], [5], [6].

Among the well-known probability distributions, the Weibull distribution established by Waloddi Weibull is a prominent probability distribution. Due to the nice physical interpretation of its parameters, the Weibull distribution is one of the most widely implemented models for analyzing real happenstances [7]. Let $\gamma \in R^{+}$ be the scale and $\phi \in R^{+}$ be the shape parameter of the Weibull distributed random variable, say $T\in R^{+}$, then, the cumulative distribution function (CDF) G(t;η) of *T* is provided by

(1) $$G\left(t;\eta \right)=1-e^{-\gamma t^{\phi }},\;\;\;\;\;\;\;\;t\in R^{+},$$

with probability density function (PDF) g(t;η) given by

$$g\left(t;\eta \right)=\phi \gamma t^{\phi -1}e^{-\gamma t^{\phi }},\;\;\;\;\;\;\;\;t\in R^{+},$$

where η=(ϕ,γ).

Undoubtedly, the Weibull model is a useful probability distribution for analyzing data having a unique-state failure rate. Unfortunately, it does not provide a competent and optimal fit when the failure rates of the datasets are in a mixed state [8]. As mentioned earlier, in most cases, the new probability distribution can provide the best fit to the actual phenomena. Therefore, considerable efforts have been made to boost and update the optimality of the Weibull distribution by adding new additional parameters [9], [10], [11], [12], [13], [14], [15], [16], [17], [18].

Most recently, Odhah et al. [19] developed a weighted cosine-*G* (WC-*G*) family with CDF

$$F\left(t;\eta \right)=\frac{e^{\left(1-\cos\left[\frac{\pi G\left(t;\eta \right)}{1+G\left(t;\eta \right)}\right]\right)}-1}{e-1},\;\;\;\;\;\;t\in R.$$

They considered its special case by taking the Weibull as a baseline distribution.

In this regard, Alghamdi abd Abd El-Raouf [20] used another trigonometric approach for studying new probability distributions with CDF

$$F\left(t;\alpha ,\eta \right)=\frac{\alpha^{\cos\left(\frac{\pi }{2}\left[1-G\left(t;\eta \right)\right]\right)}-1}{\alpha -1},\;\;\;\;\;\;t\in R,$$

where α>0 and α≠1. They also incorporated the Weibull as a special case of their suggested cosine-originated family of distributions.

Adding new parameters to the existing models may lead to re-parameterization problems. Therefore, to avoid (i) increasing the number of parameters and (ii) re-parameterization issues, we develop a novel sine-based distributional method to improve the fitting power of its special members. The proposed sine-based distributional method is called the exponent power sine-*G* (EPS-*G*) family.

Definition Assume T∈R, follows the EPS-*G* family without any additional parameters, if its CDF, say F(t;η), is

(2) $$F\left(t;\eta \right)=1-\frac{\overset{¯}{G}\left(t;\eta \right)}{e^{\sin\left[G\left(t;\eta \right)\right]}},$$

with PDF

(3) $$f\left(t;\eta \right)=\frac{g\left(t;\eta \right)}{e^{\sin\left[G\left(t;\eta \right)\right]}}\left(1+\overset{¯}{G}\left(t;\eta \right)\cos\left[G\left(t;\eta \right)\right]\right),$$

where the quantity $\overset{¯}{G}\left(t;\eta \right)$ is equivalent to 1−G(t;η).

The survival function (SF) of the EPS-*G* family, say S(t;η), is expressed by

$$S\left(t;\eta \right)=\frac{\overset{¯}{G}\left(t;\eta \right)}{e^{\sin\left[G\left(t;\eta \right)\right]}}.$$

The hazard function (HF), let express it with h(t;η), and cumulative hazard function (CHF) H(t;η) are provided, respectively, by

$$h\left(t;\eta \right)=\frac{g\left(t;\eta \right)}{\overset{¯}{G}\left(t;\eta \right)}\left(1+\overset{¯}{G}\left(t;\eta \right)\cos\left[G\left(t;\eta \right)\right]\right),\;\;\;\;\;\;\;\;t\in R,$$

and

$$H\left(t;\eta \right)=-\log\left(\frac{\overset{¯}{G}\left(t;\eta \right)}{e^{\sin\left[G\left(t;\eta \right)\right]}}\right),\;\;\;\;\;\;\;\;t\in R.$$

In Section 2, we incorporate and combine Eq. (1) with Eq. (2) to develop a special member of the EPS-*G* family. The proposed member of the EPS-*G* family is called the exponent power sine-Weibull (EPS-Weibull) model.

## 2 The EPS-Weibull model

This section defines some key functions of the EPS-Weibull distribution. Furthermore, we also provide a visual illustration of its main distributional functions.

Assume $T\in R^{+}$ follows up the EPS-Weibull model with ϕ>0 and γ>0. Its CDF F(t;η) is expressed by

(4) $$F\left(t;\eta \right)=1-\frac{e^{-\gamma t^{\phi }}}{e^{\sin\left[1-e^{-\gamma t^{\phi }}\right]}},\;\;\;\;\;\;\;\;T\in R^{+},$$

and PDF

(5) $$f\left(t;\eta \right)=\frac{\phi \gamma t^{\phi -1}e^{-\gamma t^{\phi }}}{e^{\sin\left[1-e^{-\gamma t^{\phi }}\right]}}\left(1+e^{-\gamma t^{\phi }}\cos\left[1-e^{-\gamma t^{\phi }}\right]\right),\;\;\;\;\;\;\;\;T\in R^{+}.$$

The SF of the EPS-Weibull model, say S(t;η), is expressed by

$$S\left(t;\eta \right)=\frac{e^{-\gamma t^{\phi }}}{e^{\sin\left[1-e^{-\gamma t^{\phi }}\right]}}.$$

The HF h(t;η) and CHF H(t;η) of the EPS-Weibull distribution are provided, respectively, by

$$h\left(t;\eta \right)=\phi \gamma t^{\phi -1}\left(1+e^{-\gamma t^{\phi }}\cos\left[1-e^{-\gamma t^{\phi }}\right]\right),$$

and

$$H\left(t;\eta \right)=-\log\left(\frac{e^{-\gamma t^{\phi }}}{e^{\sin\left[1-e^{-\gamma t^{\phi }}\right]}}\right).$$

**Figure 1:**
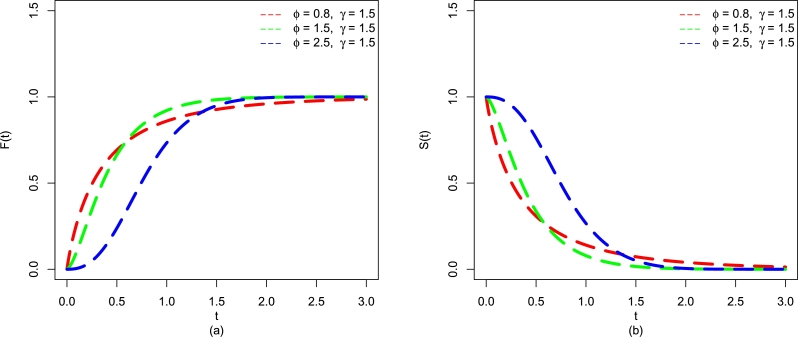
Visual illustrations of (a) F(t;η) and (b) S(t;η) of the EPS-Weibull distribution for pre-defined values of *ϕ* and fixed value of *γ* = 1.

For some pre-defined values of *ϕ* and *γ* of the EPS-Weibull model, some plots of F(t;η) (see Fig. 1(a)) and S(t;η) (see Fig. 1(b)) are presented in Fig. 1(a-b).

**Figure 2:**
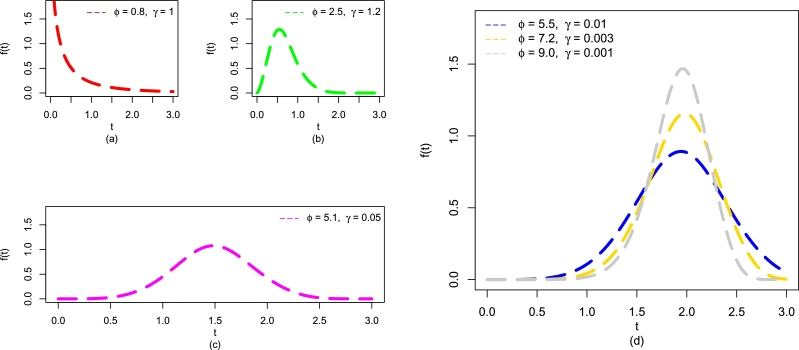
Visual illustrations of f(t;η) of the EPS-Weibull distribution for pre-defined values of *ϕ* and *γ*, including (a) decreasing, (b) right-skewed, (c) symmetrical, and (d) left-skewed.

Furthermore, some possible behaviors of f(t;η) of the EPS-Weibull distribution are obtained in Fig. 2(a-d). It shows that f(t;η) of the EPS-Weibull distribution has four distinctive behaviors such as decreasing (Fig. 2(a)), uni-modal (and right-skewed) (Fig. 2(b)), symmetrical (Fig. 2(c)), and left-skewed (Fig. 2(d)).

**Figure 3:**
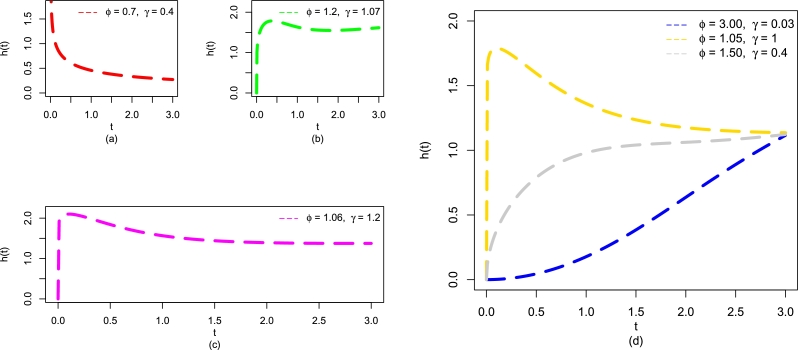
Visual illustrations of h(t;η) of the EPS-Weibull distribution for different values of *ϕ* and *γ*, including (a) decreasing, (b) modified unimodal, (c) unimodal, and (d) increasing, decreasing, unimodal.

Besides, the visual behaviors of F(t;η) (Fig. 1(a)), S(t;η) (Fig. 1(b)), and f(t;η) (Fig. 2(a-d)) of the EPS-Weibull distribution, some possible behaviors for h(t;η) of the EPS-Weibull distribution are also obtained, please see Fig. 3(a-d). The plots in Fig. 3(a-d) show that the shapes of h(t;η) can either be decreasing Fig. 3(a), increasing-decreasing (or uni-modal) Fig. 3(b), increasing-decreasing-increasing (or modified uni-modal) Fig. 3(c), and increasing Fig. 3(d). As we can see that h(t;η) of the EPS-Weibull distribution has a vary behavior offering four different pattern of the failure rate. To the best of our knowledge, there are only a few two-parameter modifications of the Weibull distribution that can offer four different patterns of the failure rate function.

## 3 Distributional properties

This part considers the investigation and derivation of certain distributional properties, especially, the quantile function (QF), median, quartile characteristics, skewness, kurtosis, $r^{th}$ moment, and moment generating function (MGF) of the EPS-*G* distributions. Here, we only provide the mathematical expressions of these distributional properties. Computer programming (or programing software), for instance, R, Python, and Mathematica can be used to obtain the numerical values of these quantities.

### 3.1 The QF

Assume T∈R follows up on the EPS-*G* family with F(t;η). Then, the QF of *T*, say $t_{q}$, is obtained by inverting F(t;η) in Eq. (4), as given by

(6) $$t_{q}=F^{-1}\left(x\right),\;\;\;\;\;\;\;\;\;0<q<1,$$

where *x* represents the solution of

sin⁡(x)+log⁡(1−q)−log⁡[1−x]=0.

### 3.2 The median and quartiles ($1^{st}$ and $3^{rd}$ quartiles)

The median (also referred as $2^{nd}$ quartile and expressed by $Q_{2}$) of the EPS-*G* family, say $t_{\frac{1}{2}}$, is

$$Q_{2}=t_{\frac{1}{2}}=F^{-1}\left(x\right),$$

or

$$\sin\left(x\right)+\log\left(1-\frac{1}{2}\right)-\log\left[1-x\right]=0.$$

The $1^{st}$ quartile (often expressed by $Q_{1}$) of the EPS-*G* family, say $t_{\frac{1}{4}}$, is

$$Q_{1}=t_{\frac{1}{4}}=F^{-1}\left(x\right),$$

or

$$\sin\left(x\right)+\log\left(1-\frac{1}{4}\right)-\log\left[1-x\right]=0.$$

The $3^{rd}$ quartile (often expressed by $Q_{3}$) of the EPS-*G* family, say $t_{\frac{3}{4}}$, is

$$Q_{3}=t_{\frac{3}{4}}=F^{-1}\left(x\right),$$

or

$$\sin\left(x\right)+\log\left(1-\frac{3}{4}\right)-\log\left[1-x\right]=0.$$

The skewness (see [21]) of the EPS-*G* family is

$$skewness=\frac{Q_{6/8}-2Q_{4/8}+Q_{2/8}}{Q_{6/8}-Q_{2/8}},$$

where the values of $Q_{6/8},Q_{4/8}$, and $Q_{2/8}$ can be obtained, respectively, by using $q=\frac{6}{8}$, $q=\frac{4}{8}$, and $q=\frac{2}{8}$ in Eq. (6).

Furthermore, the kurtosis (see [22]) of the EPS-*G* family is

$$kurtosis=\frac{Q_{7/8}-Q_{5/8}-Q_{1/8}+Q_{3/8}}{Q_{6/8}-Q_{2/8}},$$

where the values of $Q_{7/8},Q_{5/8},Q_{1/8}$, and $Q_{3/8}$ can be obtained, respectively, by using $q=\frac{7}{8}$, $q=\frac{5}{8}$, $q=\frac{1}{8}$, and $q=\frac{3}{8}$ in Eq. (6).

### 3.3 The $r^{th}$ moment

Assume T∈R follows the EPS-*G* family with PDF f(t;η). Then, its $r^{th}$ moment, say $\mu_{r}^{\prime }$, is

(7) $$\mu_{r}^{\prime }=\int_{\Omega }t^{r}f\left(t;\eta \right)dt.$$

Using Eq. (3) in Eq. (7), we obtain

(8) $$\mu_{r}^{\prime }=\int_{\Omega }t^{r}\frac{g\left(t;\eta \right)}{e^{\sin\left[G\left(t;\eta \right)\right]}}\left(1+\overset{¯}{G}\left(t;\eta \right)\cos\left[G\left(t;\eta \right)\right]\right)dt.$$

Using the series

(9) $$e^{x}=\sum_{i=1}^{\infty }\frac{x^{i}}{i!}.$$

Suppose x=sin⁡[G(t;η)] in Eq. (9), we get

(10) $$e^{\sin\left[G\left(t;\eta \right)\right]}=\sum_{i=1}^{\infty }\frac{{\left(\sin\left[G\left(t;\eta \right)\right]\right)}^{i}}{i!}.$$

Incorporating Eq. (10) in Eq. (8), we get

$$\mu_{r}^{\prime }=\sum_{i=1}^{\infty }\frac{1}{i!}\int_{\Omega }t^{r}g\left(t;\eta \right){\left(\sin\left[G\left(t;\eta \right)\right]\right)}^{i}\left(1+\overset{¯}{G}\left(t;\eta \right)\cos\left[G\left(t;\eta \right)\right]\right)dt,$$

$$\mu_{r}^{\prime }=\sum_{i=1}^{\infty }\frac{1}{i!}\left[\lambda_{1,i}\left(t;\eta \right)+\lambda_{2,i}\left(t;\eta \right)\right],$$

where

$$\lambda_{1,i}\left(t;\eta \right)=\int_{\Omega }t^{r}g\left(t;\eta \right){\left(\sin\left[G\left(t;\eta \right)\right]\right)}^{i}dt,$$

and

$$\lambda_{2,i}\left(t;\eta \right)=\int_{\Omega }t^{r}g\left(t;\eta \right){\left(\sin\left[G\left(t;\eta \right)\right]\right)}^{i}\overset{¯}{G}\left(t;\eta \right)\cos\left[G\left(t;\eta \right)\right]dt.$$

Furthermore, the MGF of the EPS-*G* distributed random variable *T*, say $M_{z}\left(t\right)$, is derived as

$$M_{z}\left(t\right)=E\left(e^{zt}\right)=\sum_{r=1}^{\infty }\frac{z^{r}}{r!}\int_{\Omega }t^{r}f\left(t;\eta \right)dt.$$

Finally, we get

$$M_{z}\left(t\right)=\sum_{r=1}^{\infty }\sum_{i=1}^{\infty }\frac{z^{r}}{i!r!}\left[\lambda_{1,i}\left(t;\eta \right)+\lambda_{2,i}\left(t;\eta \right)\right].$$

## 4 Estimation and simulation

This component part is decomposed into two parts. The first part caters the mathematical derivation of the estimators of the EPS-Weibull distribution using the maximum likelihood estimation method. While, the second subsection offers the evaluation of the maximum likelihood estimators $\left({\overset{ˆ}{\phi }}_{MLE},{\overset{ˆ}{\gamma }}_{MLE}\right)$ of the parameters (ϕ,γ) of the EPS-Weibull distribution.

### 4.1 Estimation

Assume random samples, say $T_{1},T_{2},...,T_{n}$ with observed values $t_{1},t_{2},...,t_{n}$ taken from the EPS-Weibull model with f(t;η) in Eq. (5). The likelihood function corresponding to f(t;η), say λ(ϕ,γ), is

(11) $$\lambda \left(\phi ,\gamma \right)=\prod_{i=1}^{n}f\left(t_{i};\eta \right).$$

Using Eq. (5) in Eq. (11), we get

(12) $$\lambda \left(\phi ,\gamma \right)=\prod_{i=1}^{n}\frac{\phi \gamma t_{i}^{\phi -1}e^{-\gamma t_{i}^{\phi }}}{e^{\sin\left[1-e^{-\gamma t_{i}^{\phi }}\right]}}\left(1+e^{-\gamma t_{i}^{\phi }}\cos\left[1-e^{-\gamma t_{i}^{\phi }}\right]\right).$$

Conforming to λ(ϕ,γ) in Eq. (12), the log-likelihood function, say ℓ(ϕ,γ), is

$$\ell \left(\phi ,\gamma \right)=n\log\phi +n\log\gamma +\left(\phi -1\right)\sum_{i=1}^{n}\log t_{i}-\gamma \sum_{i=1}^{n}t_{i}^{\phi }-\sum_{i=1}^{n}\sin\left[1-e^{-\gamma t_{i}^{\phi }}\right]+\sum_{i=1}^{n}\log\left(1+e^{-\gamma t_{i}^{\phi }}\cos\left[1-e^{-\gamma t_{i}^{\phi }}\right]\right).$$

With respect to *ϕ* and *γ*, the derivatives of ℓ(ϕ,γ) are delineated, respectively, by

$$\frac{\partial }{\partial \phi }\ell \left(\phi ,\gamma \right)=\frac{n}{\phi }+\sum_{i=1}^{n}\log t_{i}-\gamma \sum_{i=1}^{n}\left(\log t_{i}\right)t_{i}^{\phi }-\gamma \sum_{i=1}^{n}\left(\log t_{i}\right)t_{i}^{\phi }e^{-\gamma t_{i}^{\phi }}\cos\left[1-e^{-\gamma t_{i}^{\phi }}\right]-\gamma \sum_{i=1}^{n}\frac{\left(\log t_{i}\right)t_{i}^{\phi }e^{-\gamma t_{i}^{\phi }}\left(e^{-\gamma t_{i}^{\phi }}\sin\left[1-e^{-\gamma t_{i}^{\phi }}\right]+\cos\left[1-e^{-\gamma t_{i}^{\phi }}\right]\right)}{\left(1+e^{-\gamma t_{i}^{\phi }}\cos\left[1-e^{-\gamma t_{i}^{\phi }}\right]\right)}\;,$$

and

$$\frac{\partial }{\partial \gamma }\ell \left(\phi ,\gamma \right)=\frac{n}{\gamma }-\sum_{i=1}^{n}t_{i}^{\phi }-\sum_{i=1}^{n}t_{i}^{\phi }e^{-\gamma t_{i}^{\phi }}\cos\left[1-e^{-\gamma t_{i}^{\phi }}\right]-\sum_{i=1}^{n}\frac{t_{i}^{\phi }e^{-\gamma t_{i}^{\phi }}\left(e^{-\gamma t_{i}^{\phi }}\sin\left[1-e^{-\gamma t_{i}^{\phi }}\right]+\cos\left[1-e^{-\gamma t_{i}^{\phi }}\right]\right)}{\left(1+e^{-\gamma t_{i}^{\phi }}\cos\left[1-e^{-\gamma t_{i}^{\phi }}\right]\right)}\;.$$

Solving the functions $\frac{\partial }{\partial \phi }\ell \left(\phi ,\gamma \right)=0$ and $\frac{\partial }{\partial \gamma }\ell \left(\phi ,\gamma \right)=0$, we procure the MLEs $\left({\overset{ˆ}{\phi }}_{MLE},{\overset{ˆ}{\gamma }}_{MLE}\right)$ of (ϕ,γ) of the EPS-Weibull model.

### 4.2 Simulation

This subsection offers three simulation studies to evaluate the performance of ${\overset{ˆ}{\phi }}_{MLE}$ and ${\overset{ˆ}{\gamma }}_{MLE}$ of the EPS-Weibull model. For the EPS-Weibull model with f(t;η), random numbers are generated using Eq. (6) to accomplish simulation studies. The simulation studies are considered for five hundred iterations under different sample of sizes of n=50,100,150,200,250,300,350,400,450,500.

Using different combination values of *ϕ* and *γ*, the simulation results are obtained for:

• ϕ=0.9 and γ=1.2,
• ϕ=1.1 and γ=1.4, and
• ϕ=0.8 and γ=1.0

The performances of ${\overset{ˆ}{\phi }}_{MLE}$ and ${\overset{ˆ}{\gamma }}_{MLE}$ are evaluated using well-known statistical criteria given by

• Bias
  $$Bias\left({\overset{ˆ}{\eta }}_{MLE}\right)=\frac{1}{500}\sum_{i=1}^{500}\left({\overset{ˆ}{\eta }}_{i}-\eta \right),$$
• Mean square error (MSE)
  $$MSE\left({\overset{ˆ}{\eta }}_{MLE}\right)=\frac{1}{500}\sum_{i=1}^{500}{\left({\overset{ˆ}{\eta }}_{i}-\eta \right)}^{2},$$

**Figure 4:**
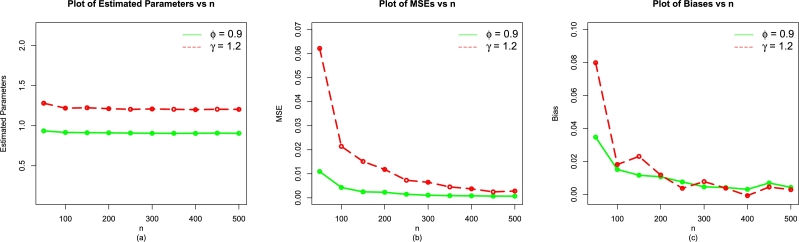
For *ϕ* = 0.9 and *γ* = 1.2, the simulation results of the (a) MLEs, (b) MSEs, and (c) Biases of the EPS-Weibull model.

**Figure 5:**
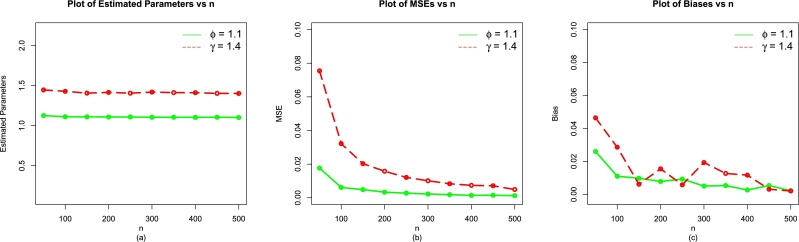
For *ϕ* = 1.1 and *γ* = 1.4, the simulation results of the (a) MLEs, (b) MSEs, and (c) Biases of the EPS-Weibull model.

**Figure 6:**
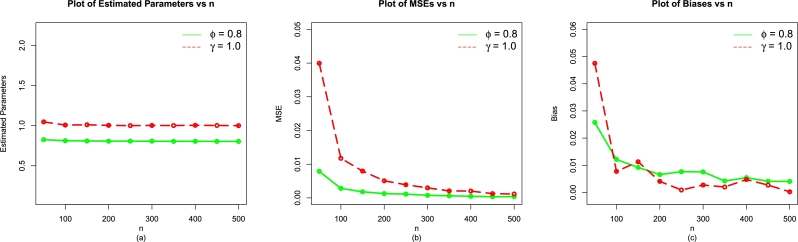
For *ϕ* = 0.8 and *γ* = 1.0, the simulation results of the (a) MLEs, (b) MSEs, and (c) Biases of the EPS-Weibull model.

The MLEs $\left({\overset{ˆ}{\phi }}_{MLE},{\overset{ˆ}{\gamma }}_{MLE}\right)$ and above evaluation criteria are obtained with the help of R software with the usage of optim(). The simulation results for the EPS-Weibull model are reported in Table 1, Table 2, Table 3 (illustrated numerically) and Figure 4, Figure 5, Figure 6(a-c) (illustrated visually).

**Table 1:** For *ϕ* = 0.9 and *γ* = 1.2, the simulation results of the EPS-Weibull model.

*n*	Parameters	MLEs	MSEs	Biases
	*ϕ*	0.9268886	0.01272384	0.036888564
50	*γ*	1.2596790	0.06225763	0.079679191

	*ϕ*	0.9166811	0.00466272	0.016681111
100	*γ*	1.2211840	0.02476091	0.021184402

	*ϕ*	0.9072911	0.00253395	0.007291090
150	*γ*	1.2098170	0.01116356	0.009816654

	*ϕ*	0.9119866	0.00221661	0.011986604
200	*γ*	1.2077050	0.00972417	0.007704685

	*ϕ*	0.9060190	0.00134644	0.006018979
250	*γ*	1.2077880	0.00634542	0.007787599

	*ϕ*	0.9053071	0.00111997	0.005307120
300	*γ*	1.2110690	0.00564636	0.011069255

	*ϕ*	0.9060128	0.00099830	0.006012752
350	*γ*	1.2018120	0.00406212	0.001812056

	*ϕ*	0.9048458	0.00079471	0.004845781
400	*γ*	1.2010620	0.00318464	0.001061710

	*ϕ*	0.9044155	0.00058226	0.004415543
450	*γ*	1.2045320	0.00284892	0.004532031

	*ϕ*	0.9049691	0.00071213	0.004969079
500	*γ*	1.2043390	0.00293717	0.004339233

**Table 2:** For *ϕ* = 1.1 and *γ* = 1.4, the simulation results of the EPS-Weibull model.

*n*	Parameters	MLEs	MSEs	Biases
	*ϕ*	1.1275900	0.01922512	0.02759045
50	*γ*	1.4609840	0.07862855	0.04898370

	*ϕ*	1.1162440	0.00830034	0.01624384
100	*γ*	1.4420430	0.03704685	0.04204346

	*ϕ*	1.1099460	0.00465996	0.00994553
150	*γ*	1.4196630	0.02088694	0.01966257

	*ϕ*	1.1095800	0.00318788	0.00957975
200	*γ*	1.4128830	0.01430159	0.01288333

	*ϕ*	1.1091470	0.00254184	0.00914668
250	*γ*	1.4146130	0.01218781	0.01461341

	*ϕ*	1.1072730	0.00218789	0.00727333
300	*γ*	1.4087790	0.01039737	0.00877877

	*ϕ*	1.1024630	0.00162482	0.00246250
350	*γ*	1.4060630	0.00785646	0.00606259

	*ϕ*	1.1060040	0.00155232	0.00600426
400	*γ*	1.4071840	0.00737316	0.00718443

	*ϕ*	1.1033510	0.00139120	0.00335062
450	*γ*	1.4053670	0.00647367	0.00536742

	*ϕ*	1.1029560	0.00115671	0.00295641
500	*γ*	1.4037330	0.00560289	0.00373320

**Table 3:** For *ϕ* = 0.8 and *γ* = 1.0, the simulation results of the EPS-Weibull model.

*n*	Parameters	MLEs	MSEs	Biases
	*ϕ*	0.8257691	0.007893175	0.025769102
50	*γ*	1.0475410	0.039965291	0.047541338

	*ϕ*	0.8121490	0.002822196	0.012149010
100	*γ*	1.0077490	0.011738299	0.007748982

	*ϕ*	0.8092015	0.001805002	0.009201524
150	*γ*	1.0112850	0.007959415	0.011285352

	*ϕ*	0.8065831	0.001265525	0.006583088
200	*γ*	1.0040830	0.005072173	0.004083323

	*ϕ*	0.8076410	0.001127473	0.007640992
250	*γ*	1.0008940	0.003893860	0.000894497

	*ϕ*	0.8075710	0.000765060	0.007571037
300	*γ*	1.0027180	0.002974502	0.002717691

	*ϕ*	0.8042253	0.000613624	0.004225287
350	*γ*	1.0020070	0.002071366	0.002007027

	*ϕ*	0.8054288	0.000473501	0.005428760
400	*γ*	1.0047570	0.002019544	0.004756642

	*ϕ*	0.8041117	0.000344468	0.004111714
450	*γ*	1.0027010	0.001250865	0.002700611

	*ϕ*	0.8040491	0.000369798	0.004049121
500	*γ*	1.0002340	0.001145137	0.000234274

As the value of *n* increases (or gets higher and higher), the numerical and visual illustrations of the simulation results of the EPS-Weibull distribution show:

• Stability of the MLEs ${\overset{ˆ}{\phi }}_{MLE}$ and ${\overset{ˆ}{\gamma }}_{MLE}$,
• decrease in the MSEs of ${\overset{ˆ}{\phi }}_{MLE}$ and ${\overset{ˆ}{\gamma }}_{MLE}$, and
• The bias of ${\overset{ˆ}{\phi }}_{MLE}$ and ${\overset{ˆ}{\gamma }}_{MLE}$ approach to zero.

## 5 Data analyses

At this part, we provide two practical illustrations (i.e., analyzing two data sets) of the EPS-Weibull model. We prove and clarify the practicality of the EPS-Weibull distribution by giving consideration to the data sets of the medical field.

### 5.1 Description of the medical data sets

At this sub-part, we present a complete description along with some basic plots of the medical data sets.

The first medical data (ahead, it may be represented by Data 1) is about the survival times of guinea pigs consisting of 72 observations. For more recent study based on the 72 guinea pigs, we refer to [23]. The second medical data (ahead, it may be represented by Data 2) is about the patients' remission times (affected by bladder cancer) consisting of 128 observations. In the recent time, researchers who considered the discussed remission times include [24] and [25].

**Figure 7:**
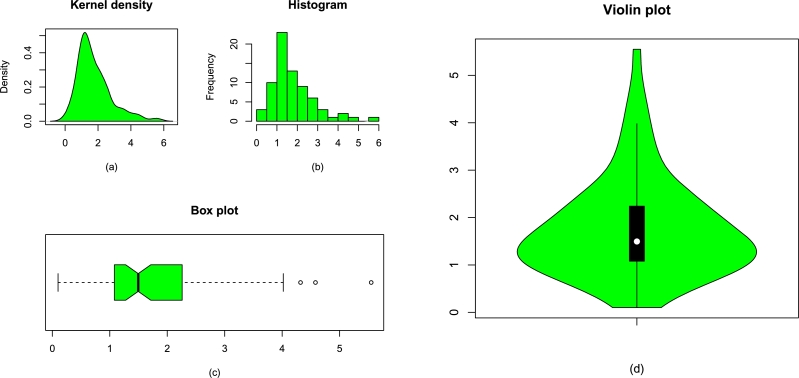
The plots for the (a) kernel density, (b) histogram of Data 1, (c) box plot of the medical data, and (d) violin plot of the medical data.

**Figure 8:**
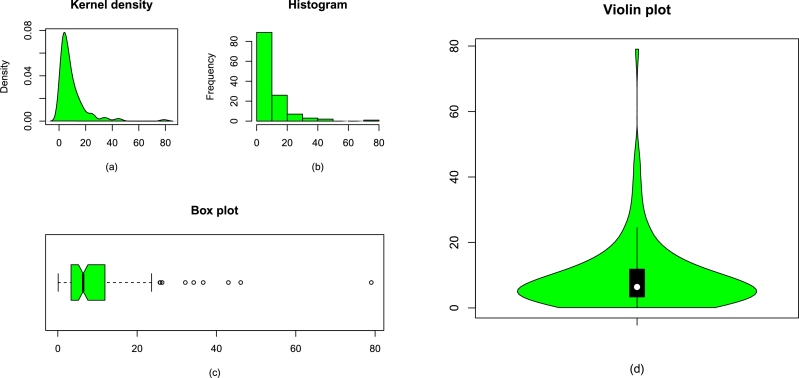
The plots for the (a) kernel density, (b) histogram of Data 2, (c) box plot of the medical data, and (d) violin plot of the medical data.

Corresponding to Data 1 (guinea pigs data) and Data 2 (remission times data), some basic plots such as the kernel density plot, box plot, histogram, and violin plot are displayed, respectively, in Fig. 7(a-d) and Fig. 8(a-d).

### 5.2 The contender distributions and selection criteria

To establish the best-suited fit of the EPS-Weibull distribution over existent models, we consider some prominent contender probability distributions. The selected contender models are:

• The two-parameter (ϕ>0,γ>0) Weibull model:
  $$S\left(t;\eta \right)=e^{-\gamma t^{\phi }},\;\;\;\;\;\;\;\;t\geq 0,\phi >0,\gamma >0,$$
• The three-parameter (ϕ>0,γ>0,β>0) new alpha cosine Weibull (NAC-Weibull) model:
  $$S\left(t;\sigma ,\eta \right)=1-\frac{\sigma e^{-\gamma t^{\phi }}}{\sigma +1-e^{-\gamma t^{\phi }}},\;\;\;\;\;\;\;\;t\geq 0,\phi >0,\gamma >0,\beta >0,$$
  and
• The three-parameter (ϕ>0,γ>0,α>0,α≠1) new extended exponential Weibull (NEE-Weibull) model:
  $$S\left(t;\alpha ,\eta \right)=\frac{\alpha^{\left(\frac{\pi }{2}e^{-\gamma t^{\phi }}\right)}-1}{\alpha -1},\;\;\;\;\;\;\;\;t\geq 0,\phi >0,\gamma >0,\alpha >0,\alpha \neq 1.$$

The optimal performances of the EPS-Weibull model and competing distributions are checked by considering seven decisive measures with the p-value. The selected decisive measures comprise of (i) four information criteria (IC) which are well-know and frequently implemented, and (ii) p-value and three quite known statistical tests. The formulas for such selected decisive measures are:

• Cramer-Von-Messes (ahead, this test is expressed by ${▿}_{1}$) test
  $$\frac{1}{12n}+\sum_{i=1}^{n}{\left[\frac{2i-1}{2n}-F\left(t_{i}\right)\right]}^{2},$$
• Anderson Darling (ahead, this test is expressed by ${▿}_{2}$) test
  $$-n-\sum_{i=1}^{n}\frac{\left(2i-1\right)}{n}\left[\log\left(1-F\left(t_{n-i+1}\right)\right)+\log F\left(t_{i}\right)\right],$$
• Kolmogorov Simonrove (ahead, this test is expressed by ${▿}_{3}$) test
  $$sup_{t}\left[F_{n}\left(t\right)-F\left(t\right)\right].$$
• Akaike IC (ahead, this IC is represented by ${▿}_{4}$)
  −2ℓ(.)+2k,
• Consistent AIC (ahead, this IC is represented by ${▿}_{5}$)
  $$-2\ell \left(.\right)+\frac{2nk}{n-k-1},$$
• Bayesian IC (ahead, this IC is represented by ${▿}_{6}$)
  −ℓ(.)+klog⁡(n),
• Hannan-Quinn IC (ahead, this IC is represented by ${▿}_{7}$)
  −2ℓ(.)+2klog⁡[log⁡(n)]

In the above mathematical formulas of the decisive measures, the terms k,n,ℓ(.), $i,F_{n}\left(t\right)$, and F(t) represent the model parameters, size of the data (i.e., sample size), LLF, $i^{th}$ sample in the data, empirical CDF, and CDF of a fitted distribution, respectively. Amidst the EPS-Weibull and contender distributions, the best-suited model with a superior fit to Data 1 and Data 2 will have the least possible values of the decisive tools and a higher p-value.

### 5.3 Analysis of the guinea pigs data

At this sub-part, we provide the real data set illustration of the EPS-Weibull that is discussed in subsection 4.1.

**Figure 9:**
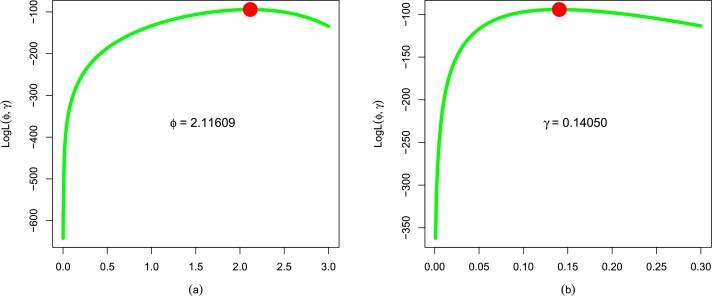
For the guinea pigs data, the log-likelihood profiles of (a) ${\overset{ˆ}{\phi }}_{MLE}$ and (b) ${\overset{ˆ}{\gamma }}_{MLE}$ of the EPS-Weibull model.

Table 4 shows the values of ${\overset{ˆ}{\phi }}_{MLE},{\overset{ˆ}{\gamma }}_{MLE},{\overset{ˆ}{\sigma }}_{MLE}$, and ${\overset{ˆ}{\alpha }}_{MLE}$ using Data 1. Using this data set, the uniqueness of ${\overset{ˆ}{\phi }}_{MLE}$ and ${\overset{ˆ}{\gamma }}_{MLE}$ of the EPS-Weibull model is established and illustrated in Fig. 9(a-b).

**Table 4:** The numerical values of ${\overset{ˆ}{\phi }}_{MLE},{\overset{ˆ}{\gamma }}_{MLE},{\overset{ˆ}{\sigma }}_{MLE}$, and ${\overset{ˆ}{\alpha }}_{MLE}$ of the fitted models or the guinea pigs data.

Dist.	$\overset{ˆ}{\phi }$	$\overset{ˆ}{\gamma }$	$\overset{ˆ}{\sigma }$	$\overset{ˆ}{\alpha }$
EPS-Weibull	2.11609	0.14050	-	-
Weibull	1.82376	0.28374	-	-
NEE-Weibull	2.34318	0.07853	0.31447	-
NAC-Weibull	1.22748	0.45063	-	13.81467

The numerical descriptions (evaluations or fitting performances) of the performances of the EPS-Weibull model and other contender distributions based on the decisive measures are shown in Table 5. For the EPS-Weibull model, we have ${▿}_{1}$ = 0.1166, ${▿}_{2}$ = 0.61401, ${▿}_{3}$ = 0.0932, p-value = 0.5580, ${▿}_{4}$ = 192.5266, ${▿}_{5}$ = 192.7006, ${▿}_{6}$ = 197.0800, and ${▿}_{7}$ = 194.3393. The results of ${▿}_{1}$, ${▿}_{2}$, ${▿}_{3}$, p-value, ${▿}_{4}$, ${▿}_{5}$, ${▿}_{6}$, and ${▿}_{7}$ in Table 5 indicate that the EPS-Weibull provides a superior and close fit to the guinea pigs' data. Furthermore, based on decisive measures and p-value recorded in Table 5, the second superior model for the guinea pigs data set is the NEE-Weibull distribution with ${▿}_{1}$ = 0.1191, ${▿}_{2}$ = 0.6590, ${▿}_{3}$ = 0.1069, p-value = 0.3821, ${▿}_{4}$ = 194.5930, ${▿}_{5}$ = 194.9460, ${▿}_{6}$ = 200.4230, and ${▿}_{7}$ = 196.3121. Similarly, the NAC-Weibull distribution and Weibull distribution stand third and fourth best models for the guinea pigs data, respectively.

**Table 5:** Using the guinea pigs data, the values of ▿_1_, ▿_2_, ▿_3_, p-value, ▿_4_, ▿_5_, ▿_6_, and ▿_7_ of the EPS-Weibull model and contender distributions.

Dist.	▿_1_	▿_2_	▿_3_	p-value	▿_4_	▿_5_	▿_6_	▿_7_
EPS-Weibull	0.1166	0.61401	0.0932	0.5580	192.5266	192.7006	197.0800	194.3393
Weibull	0.1647	0.9702	0.1051	0.4032	195.5797	195.7536	200.1331	197.3924
NEE-Weibull	0.1191	0.6590	0.1069	0.3821	194.5930	194.9460	200.4230	196.3121
NAC-Weibull	0.1565	0.9033	0.1018	0.4444	196.4686	196.8215	203.2986	199.1876

**Figure 10:**
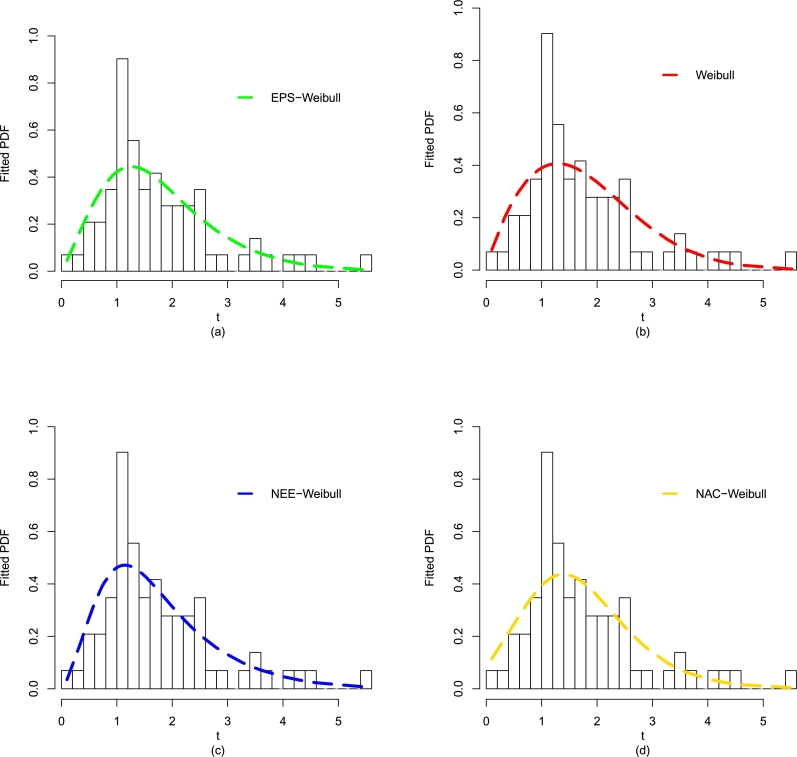
For the guinea pigs data, the visual explorations of the EPS-Weibull model and contender distributions based on the fitted PDF method.

**Figure 11:**
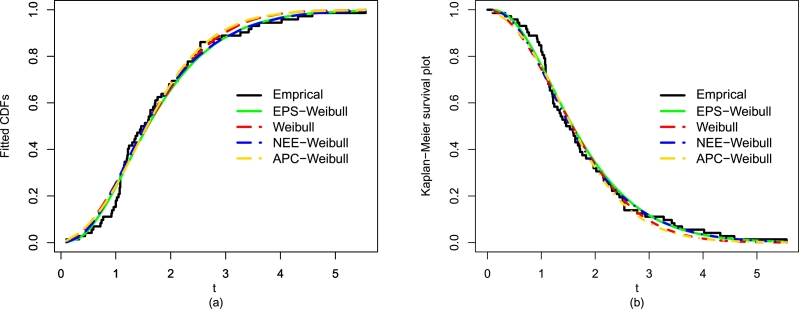
For the guinea pigs data, the visual explorations of the EPS-Weibull model and contender distributions based on the fitted (a) CDF approach and (b) SF method.

After comparing the fitting results (optimal performance) of the EPS-Weibull and its contender distributions numerically (see Table 5), now, we also provide some visual illustrations of their performances using the guinea pigs' data. The considered visual explorations are based on (i) empirical CDF approach, (ii) estimated PDF method, and Kaplan-Meier survival approach, for detail see Fig. 10(a-d) and Fig. 11(a-b). The visual illustrations in Fig. 10(a-d) and Fig. 11(a-b) also confirm that the adjustment of the guinea pigs data set by the EPS-Weibull distribution is better than rival distributions.

### 5.4 Analysis of the remission times

In this subsection, we analyze the data concerning the remission times as a second illustration of the EPS-Weibull that is discussed in subsection 4.1.

**Figure 12:**
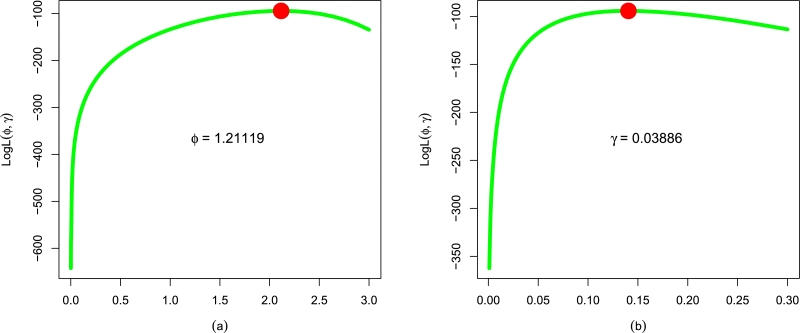
For the remission times data, the log-likelihood profiles of (a) ${\overset{ˆ}{\phi }}_{MLE}$ and (b) ${\overset{ˆ}{\gamma }}_{MLE}$ of the EPS-Weibull model.

Using the remission times data, Table 6 offers the values of ${\overset{ˆ}{\phi }}_{MLE},{\overset{ˆ}{\gamma }}_{MLE},{\overset{ˆ}{\sigma }}_{MLE}$, and ${\overset{ˆ}{\alpha }}_{MLE}$ of the fitted distributions. For this data, we again showed the uniqueness of ${\overset{ˆ}{\phi }}_{MLE}$ and ${\overset{ˆ}{\gamma }}_{MLE}$ of the EPS-Weibull distribution; see Fig. 12(a-b).

**Table 6:** The numerical values of ${\overset{ˆ}{\phi }}_{MLE},{\overset{ˆ}{\gamma }}_{MLE},{\overset{ˆ}{\sigma }}_{MLE}$, and ${\overset{ˆ}{\alpha }}_{MLE}$ of the fitted models for the remission times data.

Dist.	$\overset{ˆ}{\phi }$	$\overset{ˆ}{\gamma }$	$\overset{ˆ}{\sigma }$	$\overset{ˆ}{\alpha }$
EPS-Weibull	1.21119	0.03886	-	-
Weibull	1.05357	0.09165	-	-
NEE-Weibull	1.20118	0.04084	0.92755	-
NAC-Weibull	0.75268	0.17150	-	8.00968

After analyzing the remission times data, the evaluations (in numerical forms) of the EPS-Weibull model and contender distributions using the decisive measures and p-value are recorded in Table 7. Corresponding to the remission times data, the values of ${▿}_{1}$, ${▿}_{2}$, ${▿}_{3}$, p-value, ${▿}_{4}$, ${▿}_{5}$, ${▿}_{6}$, and ${▿}_{7}$ for the EPS-Weibull model are: ${▿}_{1}$ = 0.0692, ${▿}_{2}$ = 0.4310, ${▿}_{3}$ = 0.0580, p-value = 0.7811, ${▿}_{4}$ = 827.6687, ${▿}_{5}$ = 827.7647, ${▿}_{6}$ = 833.3728, and ${▿}_{7}$ = 829.9863. Based on the values of ${▿}_{1}$, ${▿}_{2}$, ${▿}_{3}$, p-value, ${▿}_{4}$, ${▿}_{5}$, ${▿}_{6}$, and ${▿}_{7}$ in Table 7, there is a clear indication of the superior fitting (optimal or adequately fitting) of the EPS-Weibull model to the remission times data. Based on ${▿}_{1}$, ${▿}_{2}$, ${▿}_{3}$, p-value, ${▿}_{4}$, ${▿}_{5}$, ${▿}_{6}$, and ${▿}_{7}$ reported in Table 7, the second best-suited distribution for the remission times data set is again the NEE-Weibull model with ${▿}_{1}$ = 0.0816, ${▿}_{2}$ = 0.5073, ${▿}_{3}$ = 0.0625, p-value = 0.6993, ${▿}_{4}$ = 831.1741, ${▿}_{5}$ = 831.3676, ${▿}_{6}$ = 837.7302, and ${▿}_{7}$ = 834.3505.

**Table 7:** Using the remission times data, the values of ▿_1_, ▿_2_, ▿_3_, p-value, ▿_4_, ▿_5_, ▿_6_, and ▿_7_ of the EPS-Weibull model and contender distributions.

Dist.	▿_1_	▿_2_	▿_3_	p-value	▿_4_	▿_5_	▿_6_	▿_7_
EPS-Weibull	0.0692	0.4310	0.0580	0.7811	827.6687	827.7647	833.3728	829.9863
Weibull	0.1324	0.7925	0.0742	0.4798	832.1903	832.2863	837.8943	834.5078
NEE-Weibull	0.0816	0.5073	0.0625	0.6993	831.1741	831.3676	837.7302	834.3505
NAC-Weibull	0.1248	0.7378	0.0667	0.6185	833.1293	833.3229	841.6854	836.6057

**Figure 13:**
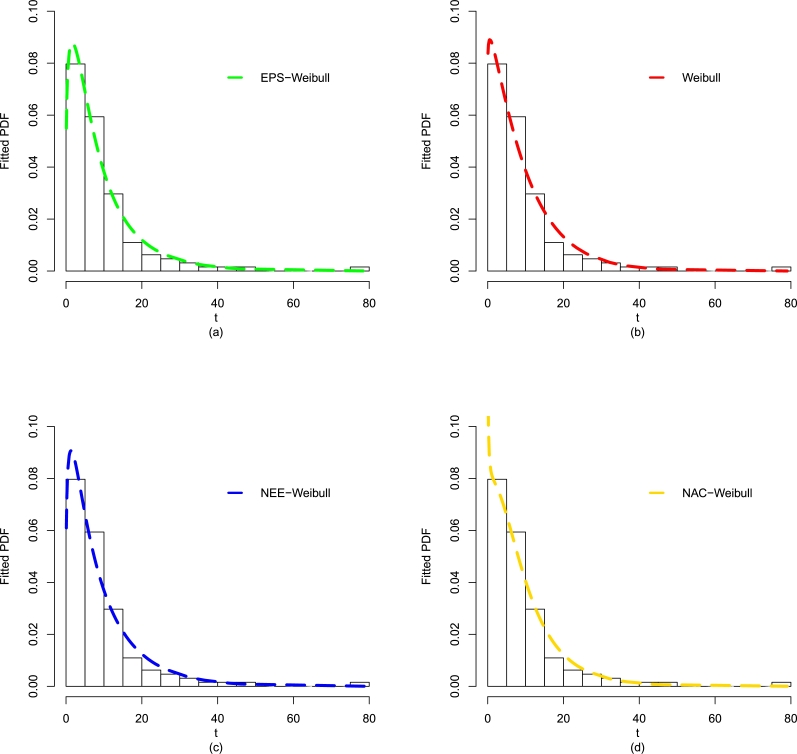
For the remission times data, the visual explorations of the EPS-Weibull model and contender distributions based on the fitted PDF method.

**Figure 14:**
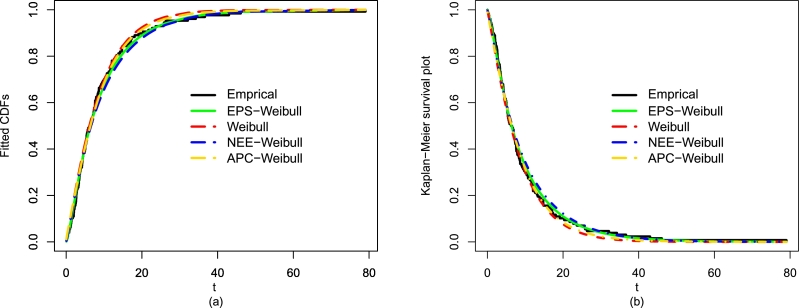
For the remission times data, the visual explorations of the EPS-Weibull model and contender distributions based on the fitted (a) CDF approach and (b) SF method.

Besides the numerical comparison using ${▿}_{1}$, ${▿}_{2}$, ${▿}_{3}$, p-value, ${▿}_{4}$, ${▿}_{5}$, ${▿}_{6}$, and ${▿}_{7}$, again we provide a graphical exploration of the EPS-Weibull model and its contender distributions. The detailed graphical exploration can see in Fig. 13(a-d) and Fig. 14(a-b). The fitted explorations in Fig. 13(a-d) and Fig. 14(a-b), again, favor the close and best adjustment of the EPS-Weibull distribution to the remission times data.

## 6 Concluding remarks

This paper takes a significant step forward by developing a new probabilistic method using the sine function. The proposed probabilistic method was called the exponent power sign-*G* family. The beauty of EPS-*G* family was introducing new probability distributions with updated flexibility levels without adding extra parameters. This advantage of the present work led to the avoidance of re-parameterization problems and reduced estimation consequences in terms of time and computational effort. Various distributional properties of the EPS-*G* family were acquired. Using the EPS-*G* method, a new useful two-parameter model, namely, the EPS-Weibull model was accomplished. The applicability of the EPS-Weibull model was demonstrated by analyzing two data sets. The first data was showing the survival times of the guinea pigs, which consisted of 72 observations. The second data was showing patients' remission times, which consisted of 128 observations. Based on 7 different selection criteria (or decision measures) with p-values, it was manifested that the EPS-Weibull model may be an adequate option for the analysis of medical data sets.

## CRediT authorship contribution statement

**Omalsad Hamood Odhah:** Writing – review & editing, Writing – original draft, Visualization, Validation, Software, Resources, Methodology, Investigation, Formal analysis, Data curation, Conceptualization. **Huda M. Alshanbari:** Writing – review & editing, Writing – original draft, Visualization, Validation, Software, Resources, Methodology, Investigation, Formal analysis, Data curation, Conceptualization. **Zubair Ahmad:** Writing – review & editing, Writing – original draft, Visualization, Validation, Software, Resources, Methodology, Investigation, Formal analysis, Data curation, Conceptualization. **Faridoon Khan:** Writing – review & editing, Writing – original draft, Visualization, Validation, Software, Resources, Methodology, Investigation, Formal analysis, Data curation, Conceptualization. **Abd al-Aziz Hosni El-Bagoury:** Writing – review & editing, Writing – original draft, Visualization, Validation, Software, Resources, Methodology, Investigation, Formal analysis, Data curation, Conceptualization.

## Declaration of Competing Interest

The authors declare no competing interests.

## Data Availability

Data included in article/supplementary material/referenced in article.
